# A roadmap to neural automatic post-editing: an empirical approach

**DOI:** 10.1007/s10590-020-09249-7

**Published:** 2020-09-03

**Authors:** Dimitar Shterionov, Félix do Carmo, Joss Moorkens, Murhaf Hossari, Joachim Wagner, Eric Paquin, Dag Schmidtke, Declan Groves, Andy Way

**Affiliations:** 1 Department of Cognitive Science and Artificial Intelligence, Tilburg University, Tilburg, The Netherlands; 2 Centre for Translation Studies, University of Surrey, Surrey, UK; 3 ADAPT Centre and School of Applied Language and Intercultural Studies, Dublin City University, Dublin, Ireland; 4 ADAPT Centre, School of Computing, Dublin City University, Dublin, Ireland; 5 Microsoft, South County Business Park, Leopardstown, Dublin, Ireland

**Keywords:** Automatic post-editing, Neural post-editing, Multi-source, Deep learning, Empirical evaluation, Machine Translation

## Abstract

In a translation workflow, machine translation (MT) is almost always followed by a human post-editing step, where the raw MT output is corrected to meet required quality standards. To reduce the number of errors human translators need to correct, automatic post-editing (APE) methods have been developed and deployed in such workflows. With the advances in deep learning, neural APE (NPE) systems have outranked more traditional, statistical, ones. However, the plethora of options, variables and settings, as well as the relation between NPE performance and train/test data makes it difficult to select the most suitable approach for a given use case. In this article, we systematically analyse these different parameters with respect to NPE performance. We build an NPE “roadmap” to trace the different decision points and train a set of systems selecting different options through the roadmap. We also propose a novel approach for APE with data augmentation. We then analyse the performance of 15 of these systems and identify the best ones. In fact, the best systems are the ones that follow the newly-proposed method. The work presented in this article follows from a collaborative project between Microsoft and the ADAPT centre. The data provided by Microsoft originates from phrase-based statistical MT (PBSMT) systems employed in production. All tested NPE systems significantly increase the translation quality, proving the effectiveness of neural post-editing in the context of a commercial translation workflow that leverages PBSMT.

## Introduction

Machine Translation (MT) is widely employed in industrial translation workflows. MT for dissemination is an intermediate step which generates a raw translation of a given source document or a sentence, followed by a post-editing step that ensures that the quality of the final translation meets required quality standards. Automatic Post-editing (APE) is an area of research aiming at exploring methods that apply editing operations on an MT output to produce a better translation and thus reduce the human effort in the translation workflow.

APE covers a wide range of post-editing approaches, from regular expressions applied on the MT output to post-editing simple error patterns, to deep learning techniques that can transform complete sentences, paragraphs or even documents into a more correct variant. Needless to say, while APE aims to reduce certain MT errors, it is up to the human translator to accept or further post-edit the output. In this article, we focus on APE with deep neural networks—neural APE or simply neural PE (NPE)—and the sentence-to-sentence post-editing case.

In the rest of this article we use the following abbreviations and notations:

- **SRC** segment(s) in the source language;
- **MT** the output of a non-specified MT system;
- **PE** the version of the MT segment(s) after post-editing by professional translators;
- **SMT** the output of an SMT system, usually the baseline;
- **NMT** the output of an NMT system;
- **NPE** the version of the MT segment(s), after post-editing with an NPE system;
- **TER(npe, pe), TER(smt, pe)** the TER score between NPE or SMT (the hypothesis) and PE (the reference), a human post-edited version of the machine translation output is used as reference.^1^

### Automatic post editing and its parallelism with machine translation

APE systems convert a segment *e* in the target language *L*2 to a *corrected* variant $e'$ in the same language. The APE task can be seen as a monolingual translation task where the source and the target language are the same. As such, APE implementations are rather similar to MT systems and even employ similar methodologies. However, while in an MT scenario a system is trained on pairs of sentences (*f*, *e*) in two different languages, in an APE scenario the available data includes MT input and output, as well as the human post-edited variant of it. That is, an APE system is trained on triplets of sentences—$(f, e, e')$—where $e'$ is the post-edited variant of *e*. Such triplets (i) reveal the transformations of *e* into $e'$ that should be learned by the APE, for it to correct automatically any new data and (ii) allow for a consistency check with the source sentence *f*. The learning process depends on the availability of enough triplets.

### Data demands and data scarcity

While the collection of parallel data (for training MT engines, for example) has been an ongoing process since the beginning of SMT, APE is a recently-emerged approach. For many language pairs, thus, enough parallel data is available. However, that is not the case for triplets with human post-edited data, required in data-driven APE approaches.

To mitigate this issue for the open development of APE systems, datasets of artificially generated triplets have been produced and made publicly available. In Junczys-Dowmunt and Grundkiewicz (2016) one such set for the English → German language direction is described. It is generated via round-trip translations using two PBSMT engines, one for the German → English and another for the English → German language directions. The synthetic post-edit triplets are composed of the German source data as the post-edited data, the German → English translated data as the English source, and the round-trip translation output as the uncorrected MT data. Consecutively, the data is filtered according to TER to mimic the quality of the provided APE data. More recently, Negri et al. (2018) present the eSCAPE corpora covering multiple language pairs. Their method is to translate freely-available data and use the target side as a human post-edited version of this translation, thus creating triplets of sentences.

Exploiting synthetic data has shown to lead to improvements in APE (Bojar et al. 2017; Chatterjee et al. 2018) and in NMT systems (Sennrich et al. 2016b; Poncelas et al. 2018). A detailed summary is presented in do Carmo et al. (2020). However, Poncelas et al. (2018) show that using excessive synthetic (i.e. backtranslated) data can lead to deterioration of quality. Nevertheless, in an industry environment where human post-editing is a standard procedure, a sufficient quantity of closed-access triplet data is often available (Crego et al. 2016; Mathur et al. 2017). This was the case in the collaborative project between the ADAPT centre and Microsoft described in this article. This data is typically (i) optimised towards the domain of application (i.e. with respect to terminology, style, etc.) and (ii) conforms with the quality standard requirements.

In this article, we present NPE systems trained on industry-standard data for the English–German (EN–DE) and English–Spanish (EN–ES) language pairs. And while the data we used is not publicly available, this article aims to convey our knowledge and experience on such data, making it easier for researchers to understand industry requirements and to provide solutions that apply not only in academic but also in commercial conditions.

### The ADAPT-microsoft APE project

The systems we present in this article are the result of a collaboration between the ADAPT Centre and the Microsoft GSX Language Technology group that took place between May and September 2018.

This collaborative project aimed to test the use of NPE in a commercial environment and with industry-standard data. The data, provided by Microsoft, is part of the production data. Within the scope of this project, we considered two language pairs: English–German (EN–DE) and English–Spanish (EN–ES), exploited in two rounds.

The project was divided into two main stages: *stage i* State-of-the-art review and analysis, and *stage ii* Implementation and Empirical evaluation.

In *stage i* we conducted a review of the state-of-the-art of APE systems. The purpose of this first stage was to inform the empirical one, i.e. *stage ii*, and guarantee that the best technology available was employed for the purposes of the project. A summary and analysis of state-of-the-art APE systems is presented in do Carmo et al. (2020).

*Stage ii* started with a full analysis of the data provided by Microsoft. Section 3 of this article summarises our findings with respect to the data. This analysis allowed us to identify specific features in the data that conformed the models tested at the next stage.

The implementation and evaluation part was divided in two. First, we trained and evaluated NPE systems with EN–DE data. This round ran as a standalone project encompassing data analysis, data preprocessing, deciding on NPE systems to train, and training and assessment, all using only EN–DE data. Based on the results, observations and acquired knowledge from the first round, we conducted a second set of experiments with EN–ES data. That is, we selected some of the best approaches and we ran experiments on EN–ES data, with fewer systems involved. Overall we trained 15 different systems (11 systems with EN–DE data and 4 with EN–ES data), exploring diverse setups with and without augmentation of the input data. These systems are described in Sect. 4.

In the evaluation part we collected standard edit scores—TER (Snover et al. 2006) and BLEU (Papineni et al. 2002)—of the different systems under comparison. A detailed analysis of the results was also performed. Our evaluation results are reported in Sect. 5.

## Neural APE

In this work, we analyse and empirically evaluate different NPE approaches and present the most efficient ones. The ultimate goal of this work is to inform the reader about the end-to-end process for achieving high-quality APE output, along with the conditions and limitations of the various approaches. In addition, we exploit industry data composed of triplets ($\{src, mt, pe\}$) where the post-edited segments originate from professional translators. Thus, we aim to draw a roadmap over existing neural APE techniques. The major decision points are related to (i) the neural architecture and (ii) how the data is used for training the NPE system. We do not investigate the effects of adapting low-level settings of the neural systems, such as the learning optimiser, the size of the neural networks, etc. as they are outside of the scope of this project; we consider the default settings to be effective for our tasks. In (Junczys-Dowmunt and Grundkiewicz 2017) an analysis of various sequence-to-sequence architectures for APE is presented, over the differences and the effects of the various attention mechanisms on APE quality. Our work aims to explore various architectures in a commercial environment and it focuses on the dependencies between data and model architectures.

As noted in Sect. 1, the APE task involves handling multi-source input and a single source output (*Input*: $\{src, mt\}, Output$: *pe*). In a sequence-to-sequence encoder-decoder architecture, the multi-source input can be handled with either a single encoder or with multiple encoders. That would impose different requirements towards preprocessing the data and building dictionaries. For example, if a single encoder is used, then the SRC and MT need to be concatenated and a joint dictionary needs to be built.

A double-encoder NMT model, where SRC and MT inputs are encoded separately and the corresponding context vectors are used together as input to the decoder, would require separate dictionaries: two for the SRC and MT and one for the PE data. In the latter case the dictionary size would be smaller than the one of a joint dictionary in the former (single-encoder) case.

Another decision on how to approach NPE is whether to use ensembles of models or a single-model NMT system. To handle different types of input/output pairs (e.g. based on character count or word count) we can either implement an ensemble NMT system, where different networks will be trained on the different input/output pairs, or one single network trained on data carrying extra information regarding its type. In this case, a prefix token can be added to each input pair or triplet, which identifies the type(s) of input. While ensembling is widely used for NMT, quality estimation, and APE, it complicates the architecture, adding an extra layer of training and optimisation. This type of engineering overhead makes it prohibitive for large-scale QE employment that is required in a commercial workflow. For example, Microsoft operates with more than 80 languages, therefore it is easy to see how important it is to choose an efficient and scalable approach for production. The latter approach, i.e. adding a prefix token to the input sequence, follows from transfer learning and has been employed successfully on multi-language MT (Johnson et al. 2017; Mattoni et al. 2017), gender identification in MT (Vanmassenhove et al. 2018) and controllability in MT (e.g., to manage forms of politeness (Sennrich et al. 2016a)). Using a prefix token allows only one system to be trained jointly to perform APE on different types of input. To our knowledge, at the time of conducting the experiments, our work was the first to employ such an approach for APE.

For NMT, splitting words into subword units has led to state-of-the-art results. The most common method is unsupervised Byte Pair Encoding (Sennrich et al. 2016c), BPE in short, a fast and language-independent method. Other methods based on morphology (e.g. based on Morfesor (Creutz et al. 2005; Smit et al. 2014)) have also led to good results for specific languages (Ataman and Federico 2018). Their main drawback is the language dependency. Segmenting tokens into their basic building blocks, i.e. characters, has been explored for character-based NMT (Lee et al. 2017) and also for character-based APE (Junczys-Dowmunt and Grundkiewicz 2017). For our NPE systems, we considered words and subword units generated with BPE. Increasing the granularity of subword units to characters would imply that the APE system would have to learn how to correct the spelling of specific words—a task that is computationally more expensive and unnecessary for our use case (the dataset originates from SMT systems and it is therefore expected to be correctly spelled).

Another decision point is whether to extend the training data with data-specific features. Similar to Hokamp (2017), an option is to add syntactic features as factors and train a factored NMT system.

**Fig. 1:**
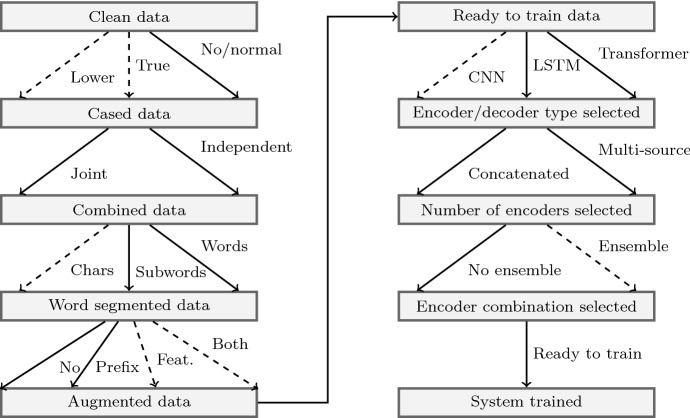
A roadmap over the decision points for an APE system. Each node represents a stage prior to training an APE system. Each transition between the nodes on the map represents the different options of a single decision point. A transition between two nodes should follow only one of the available edges, that is, multiple options for a single decision point are not possible. Continuous lines indicated options we empirically evaluated in Sect. 4; for the rest we present thorough analysis based on literature and preliminary experiments. Legend (following the roadmap): *Lower* lower-cased data; *True* true-cased data (only change words at the beginning of a sentence to their most frequent form); *No/normal* do not apply any casing transformation; *Joint* combine SRC, MT and PE data to build a joint vocabulary or word-segmentation (e.g. BPE); *Independent* do not combine SRC, MT and PE data, but apply consecutive transformations independently on each of them; *Chars* segment each word on character level; *Subwords* segment each word on subword level (e.g. after BPE); *Words* no word segmentation; *No* no data augmentation; *Prefix* data is augmented using a prefix on each sentence; *Feat.* per-word features; *Both* prefix and features; *CNN* NMT with CNN-based encoder and decoder; *LSTM* NMT with LSTM-based encoder and decoder; *Transformer* Transformer NMT architecture; *Concatenated* SRC and MT input sentences are concatenated; *Multi-source* SRC and MT are fed separately into an encoder; *No ensemble* do not ensemble models; *Ensemble* ensemble models

These different decision points are mapped in Fig. 1 in the form of a *roadmap*. We follow this roadmap to systematically construct our experiments and train the corresponding systems. Such a systematic empirical evaluation aims to inform the reader of the possible options and their implications in constructing APE systems for other use cases.

## Data analysis

The data provided by Microsoft (actual production data), constitutes a collection of 201,000 triplets, translated from English into two different target languages: German (EN–DE) and Spanish (EN–ES). These triplets include the source English string (SRC), its machine translation (MT)—originating from an SMT system—in different moments in time, and their corrected versions created by human post-editing (PE). The data consisted of user interface (UI) strings, that is, it contained menu entries, help messages, etc. from different Microsoft software products.

The following analysis was produced only for the EN–DE language pair, during the first round of *stage ii* of the project.

### Pre-processing

First, we performed a data analysis on the English-German data to identify issues and irregularities that might impede the performance of an APE system. We investigated strings such as *untranslatable items*, *file names/paths/locations*, *hyperlinks*, *markup*, *structured alphanumericals*, and so on, which are very frequent in UI data. The inappropriate handling of these strings, e.g., incorrect tokenisation, could trigger an APE system to post-edit an already correct translation, i.e. the problem of overcorrection. As a result, we proposed and implemented a pre-processing step to clean and normalise the data. This step included the normalisation of spaces, punctuation, quotes, and other special characters.

Segment duplication was also analysed. It is a typical situation in production scenarios, where the same segment may be produced in different projects and translated repeatedly. We identified 0.4% of the data as full duplicates—same SRC, MT and PE—and removed them.

We also noted that some segments in the provided data form small paragraphs, containing more than one sentence. We analysed the distribution of such segments and identified that 22% of the segments contained more than one sentence, and only 0.4% of the segments contained more than five sentences. We assessed their structure and found it impractical to segment these to the sentence level. It is typical in NMT and APE to cut sentences to around 60 tokens for efficiency and performance purposes. To accommodate these long sentences, we extended this cut-off limit to 300 and 150 tokens, for concatenated and multi-source systems respectively (see Sect. 4).

We implemented a pre-processing step that tackles the aforementioned issues and further cleans the data. While some of the pre-processing is language independent, e.g., file names/paths/locations, other modifications are language dependent. In Sect. 4.1 we discuss the employment of this step in our experiments for the EN–DE data.

### Partitions

Next, we analysed the data with respect to different common characteristics that might show similar patterns and potentially guide the APE systems. Besides the triplets of *SRC*, *MT*, *PE*, the provided dataset included information such as the software package that was translated, the translation project, a timestamp and other metadata. We hypothesised that each of these metadata categories could act as a factor in grouping the data into partitions that share common features. These common features in turn would help the decoding process to find a better post-edited candidate. We studied several ways to partition the data, using a criterion of relevance, based on the distribution of data in the classes, to decide which would be used in the experiments. Following, we present the main partitions we considered together with the factors and reasons for focusing on these.

- *Length:* Microsoft provided a word count of each source segment, produced with Microsoft’s internal tools. Microsoft wanted to receive results and observations for subsets of the data based on the following length intervals: (i) 0–4; (ii) 5–9; (iii) 10–30; (iv) 31–$\infty$. We distributed the data into 4 partitions based on the length of the source segments: $Len_1, Len_2, Len_3, Len_4$.
- *Tenant:* another useful metadata label is the tenant description. A tenant is a grouping of projects, according to Microsoft organisation. We used this information to form 14 partitions of our data according to the *tenant* label.
- *TenantPartition:* because some partitions based on the tenant label contained a very small number of segments, we further organised the data into 6 partitions—the top five tenants as specific partitions, and the others into one single partition called “Other”. In the rest of this article we refer to this partitioning as *“TenantPartition”*.

Aside from these three classes for partitioning the data, we also considered *Project* and *Number of sentences in a segment* as relevant criteria, in addition to different ways to calculate word and token counts. However, these were not considered relevant due to the unbalanced way in which segments were distributed according to these criteria. For example, there was a high number of projects (583), many of which contained a small number of segments:

- The largest project, “DevSuitePortal”, had 16.5k segments (8.2%).
- The second largest, “word-Office-ios”, had 9.6k segments (4.8%).
- The top 5 cover 47.9k (23.8%), the top 10 cover 73.2k (36.4%).
- 350 projects had less than 100 segments.
- 94 projects had less than 5 segments.

As for the number of sentences in a segment, we analysed their distribution, and we identified that 88% of the segments contained 1 sentence, and there were only 0.4% segments with more than five sentences. Due to the skewed distribution, we decided not to use this as an informative feature for data partitioning.

### Editing patterns

A brief analysis of some of the editing patterns in the data was also done at this stage, although it was not intended to apply these as components of the training systems. A type/token ratio analysis and an analysis of unique trigrams showed that PE sentences had a richer vocabulary, which was used more consistently than in MT output. These data conform with the findings presented in (Vanmassenhove et al. 2019) and Toral (2019) about the differences the lexical richness between human and machine translated text.

We also observed that in 27.4% of the segments the MT output had not been post-edited. A close analysis of some of these segments shows segments composed of placeholders, numbers, URLs, or other non-translatable elements. The number of segments in which the PE content is the same as the SRC content is around 10%, again with some cases of untranslatable elements or placeholders.

The distribution of editing operations observed in the training data was as follows: a higher number of substitutions (22%), followed by deletions (15.4%), with insertions and shifts at similar proportions (ca. 4%). This distribution is typical of PE scenarios and it is more or less reproduced by the best APE systems (do Carmo et al. 2020).

### Training, development and test data

The provided data consisted of 180,198 triplets of segments as training data (SRC,SMT → PE), 10,000 triplets as test and 10,000 as development sets. For the two language pairs the source side of the training data is the same, however the test and development sets are different, since the sampling method did not only use features of the source segments, but also features of the target segments.

Our data analysis also guided the selection of the development and test sets. We took into account the main features and partitions identified in the data. The features that were considered relevant for extracting a balanced sample of the dataset were: length of the segment (source words), token count of the PE data, the tenant, the TER scores estimated between MT and PE, and number of sentences in a segment. We selected randomised and stratified subsets for the training, development, and test sets.

For both training and translation, we use tokenised, normal-cased sentences. We applied neither lowercase nor truecase to the tokens, but used their original form, in order to account for casing errors. By using original-case data, the NPE system would learn to recognise casing errors we aim to fix along with everything else.

## Experiments setup and tested systems

We followed the roadmap of Fig. 1 and built 15 NPE systems, alternating between the different options on the choice points. We first trained and evaluated 11 systems for the EN–DE language pair. Following their evaluation, we used the parameters that led to the best performance to train 4 systems for the EN–ES language direction. Our empirical assessment then aimed to: (i) identify the best system for our use case and for the two language pairs and (ii) identify how different system variables affect the NPE performance.

### Systems

*Pre-processing:* Following the discussion in Sect. 3 with respect to the EN–DE data, we added an extra decision point on our roadmap regarding the *pre-processing* of the data. There were two pre-processing methods—we could either use the original data as preprocessed by Microsoft, or the data that resulted from applying ADAPT’s pre-processing which fixes spaces, quotes, and other issues as presented in Sect. 3.1. That gave rise to two different types of systems.

We ought to note that while the ADAPT pre-processing led to better results (see later in Sect. 5) we decided to use Microsoft’s (pre-processed) data in the second experiment round with EN–ES data. The reason is two-fold: (i) a lot of the pre-processing is language dependent and (ii) the observed improvements are not big enough to justify the manual labour required to identify a good pre-processing procedure for the EN–ES data.

*Tokens and dictionaries* The choice for word segmentation granularity impacts not only the data vocabulary (i.e., the system’s dictionary), but also the choice of Encoder/Decoder. We considered three different strategies to build dictionaries: (i) Character-based; (ii) BPE, including 50k BPE operations; (iii) Word-based. For different use cases, each of these methods has been shown in literature to have a positive impact on the translation quality, and under different conditions it can be preferred to the others. While in a post-editing scenario it is important to learn how to correct complete words, rather than sub-word particles (characters or BPE-based subwords) an important shortcoming of word-based dictionaries is the large vocabularies that, if reduced for the model to fit in memory, may result in out-of-vocabulary (OOV) issues. At the other extreme, using characters as basic tokens implies long sequences that are hard to process (from time and resource perspectives and diminishing performance for LSTM models) with sequence-to-sequence models (Pascanu et al. 2012). To target this issue, convolutional neural networks (CNNs) have been successfully employed in MT (Lee et al. 2017) and APE (Varis and Bojar 2017). In this work we aim to address the performance of the more mainstream LSTM and Transformer models.

In our experiments we look into BPE- and word-based dictionaries.

*Data augmentation* At this stage we had to decide whether and what extra input information to add. In Sect. 3.2 we identified several data partitions based on certain properties of the input/output data. We consider the three partitions—(i) Length, (ii) TenantPartition and (iii) Tenant—as most characteristic and use them to augment our data. To do so, we introduce an extra token in front of the input sequence that states the partition it belongs to. We refer to systems trained with extra information about the Length, TenantPartition and Tenant as Augmented 1, Augmented 2 and Augmented 3 accordingly (see Tables 1 and 2).

Similar to Hokamp (2017) we also explored features based on part-of-speech (POS) tags and dependency parses. However, our preliminary experiments showed that in a scenario where the SRC and/or MT segments do not constitute well-formed sentences, as is the case of the UI data in our use case, adding such features impedes the performance of the system. Our results were below acceptable and after the preliminary tests, we discontinued experimenting with word-level linguistic features and focused on the prefix-token augmentation. We ought to note that while our results in the specific use case discard linguistic features, other types of word-level features may contribute to the overall performance. However, this reaches out of the scope of our work and we did not pursue this direction.

*Input representation* Given that the input consists of two types of sequences—the SRC and the SMT—we can choose between (i) Single sequence input where SRC and SMT are concatenated or (ii) Multi-sequence input where SRC and SMT are fed separately. The former case would imply the use of one encoder, while for the latter, two separate encoders—one for the SRC and another for the SMT sequences.

Furthermore, in the single sequence case, it is important to consider the order in which sentences are presented—either the SRC is the first part of the sequence followed by the SMT, or the other way round. We used concatenated input, i.e. single sequence input, with the SRC being the first part of the input. We also tested multi-sequence input, where SRC and SMT are provided as two different inputs to a multi-encoder architecture.

*Ensembles* As justified in Sect. 2, we do not explore system combination with ensembles. In our exploration we focus on single-model systems only.

Based on these variables, we implemented a set of APE systems using the Marian-NMT toolkit. The set of tested systems contains 8 *Vanilla* systems (when we only train with the provided language data and we test different NMT implementations) and three *Augmented* systems (when extra information is added to the data). This strategy allowed us to incrementally test characteristics from different state-of-the-art systems, and to explore knowledge retrieved during the data analysis, as presented in Sect. 3. Table 1 summarises the systems and the system options we trained for the EN–DE language pair.

**Table 1:** NPE systems and the choice of variable options for the EN–DE language pair

System	Preprocessing	Word-segment	Input representation	Encoder	Extra information
Vanilla 1	ADAPT	BPE	Concatenated	LSTM	No
Vanilla 2	Microsoft	BPE	Concatenated	LSTM	No
Vanilla 3	ADAPT	Words	Concatenated	LSTM	No
Vanilla 4	Microsoft	Words	Concatenated	LSTM	No
Vanilla 5	ADAPT	BPE	Multi-source	LSTM	No
Vanilla 6	Microsoft	BPE	Multi-source	LSTM	No
Vanilla 7	ADAPT	BPE	Multi-source	Transformer	No
Vanilla 8	Microsoft	BPE	Multi-source	Transformer	No
Augmented 1	ADAPT	BPE	Multi-source	LSTM	LENGTH
Augmented 2	ADAPT	BPE	Multi-source	LSTM	TENANTPartition
Augmented 3	ADAPT	BPE	Multi-source	LSTM	TENANT

After training and assessing the performance on the EN–DE data, we selected options and trained 4 systems for the EN–ES language pair. These are listed in Table 2. While evaluation scores proved that ADAPT’s preprocessing improves the performance of the EN–DE systems, we do not employ such preprocessing for the EN–ES systems as discussed earlier in this section.

**Table 2:** NPE systems and the choice of variable options for the EN–ES language pair

System	Preprocessing	Word-segment	Input representation	Encoder	Extra information
Vanilla 1	Microsoft	BPE	Concatenated	LSTM	No
Augmented 1	Microsoft	BPE	Multi-source	LSTM	LENGTH
Augmented 2	Microsoft	BPE	Multi-source	LSTM	TENANTPartition
Augmented 3	Microsoft	BPE	Multi-source	LSTM	TENANT

### System setup

We trained our systems on an Intel-CPU (Intel(R) Core(TM) i7-5960X CPU @ 3.00GHz) machine, with two Titan X GPU cards with 12GB RAM each. The machine itself has 64GB of RAM. We used one GPU for each system (training and translation).

Our training and translation pipelines (see below, after the list of setup options) are written in bash and invoke scripts in the following languages: Python 3.6, Java, and Perl. The version of MarianNMT that we used was 1.5.0 and the setup was as follows:

- *Options* –mini-batch-fit, –workspace 9000, –layer-normalization, –dropout-rnn 0.2 –dropout-src 0.1 –dropout-trg 0.1, –early-stopping 5, –max-length 150 –max-length-crop, –valid-freq 2000 –save-freq 2000 –disp-freq 1000.
- *Validation metric* cross-entropy translation.
- For multi-source systems the max-length was 150, while for the concatenated systems, the max-length was double the size, i.e. 300.

We used the amun decoder for the concatenated systems, the multi-s2s and the multi-transformer for the multi-source systems.

### Experiment pipeline

We implemented a pipeline of 5 processing steps. Given that the data provided is already tokenised, split into train, test and development sets and pre-processed, no pre-processing and tokenisation were executed in the experiments pipeline. One exception is the invocation of a step to concatenate the two input sequences into a single one, for the *Vanilla 1*—*Vanilla 4* systems, trained only in the first round (EN–DE). The 5 processing steps of our pipeline are described next, together with the general settings.

1. *Create dictionaries* To retain language independency, we built our dictionaries on source and target languages independently. In particular, given the SRC, SMT, and PE triplets, the first step will create 3 dictionaries. Prior to extracting each of these dictionaries, byte pair encoding (BPE) is applied on the data to create sub-words which will constitute our dictionaries. Dictionaries are extracted only from the training data, i.e., test and development sets are not considered when creating the dictionaries.
2. *Train* Train an NPE system given the two source datasets (SRC and SMT) and the PE as a target set. These were the training options:
  Use development set for validation.
  Use cross-entropy as the validation and stopping criteria. We used cross-entropy rather than BLEU to limit the chances to overfit on the development set.
  Define early stopping after 5 updates that do not improve the cross-entropy.
  Compute BLEU on the validation set for each 2000 updates.
  Save an intermediate model each 2000 updates.
  Set workspace memory to 9 GB: given that our GPUs have memory of 12 GB, we allowed for 9GB to be used during training; the rest is used during validation.
  Use the –mini-batch-fit option and do not explicitly indicate batch size. Due to the differences in our systems, we did not explicitly set the batch size and we allowed MarianNMT to determine optimal sizes for each batch. In some cases, setting the batch size to 64, while using word-based dictionaries, would lead to out-of-memory issues.
  We also used layer normalisation and dropout.
  Set maximum length of the input sequences for concatenated systems (*Vanilla 1*—*Vanilla 4*) to 300; for all other systems, it was set to 150.
3. *Translation* After a system is trained, it can be invoked to translate text. In the case of concatenated systems, the input is one file that contains tokenised sentences in the source language, concatenated to sentences in the target language (that are the output of the MT system). For the multi-source systems, there are two separate inputs—one is the SRC data and another is the SMT data. The generated output file contains tokens with BPE-specific characters that need to be removed in the next step.
4. *Post-processing* BPE-specific characters are removed and the sequences are detokenised.
5. *Evaluation* Our pipeline contains a script for scoring the output quality against the PE part of the triplets that formed the test set. The script uses two options for BLEU and one for TER: (i) Moses BLEU is the multi-bleu.pl script distributed with Moses 2.1; (ii) MultEval BLEU is the version of the algorithm as implemented within the MultEval tool; (iii) TER is as implemented within the MultEval tool. This evaluation process with multiple metrics allowed us to better assess and compare the quality of the systems.

### Vocabulary sizes

The vocabulary sizes for the concatenated systems for the EN–DE and EN–ES language pairs are shown in Table 3.

**Table 3:** Vocabulary sizes for systems with concatenated input

Language pair	Preprocessing	Word-segmentation	SRC $\cup$ SMT	PE
EN–DE	ADAPT	BPE (50*k* operations)	44,796	41,631
EN–DE	Microsoft	BPE (50*k* operations)	44,820	41,614
EN–DE	ADAPT	Words	77,924	58,080
EN–DE	Microsoft	Words	78,834	58,181
EN–ES	Microsoft	BPE (50*k* operations)	44,865	41,769

For the multi-source systems, we used only BPE-based vocabulary, since using word-based vocabularies would lead to memory outage. The vocabulary sizes are shown in Table 4.

**Table 4:** Vocabulary sizes for systems with multi-source input

Language pair	Preprocessing	Word-segmentation	SRC	SMT	PE
EN–DE	ADAPT	BPE (50*k* operations)	39,024	40,950	42,246
EN–DE	Microsoft	BPE (50*k* operations)	39,570	40,878	42,200
EN–ES	Microsoft	BPE (50*k* operations)	37,473	40,841	41,769

A partition token for Augmented 1 to Augmented 3 systems is additionally added to the vocabulary.

### Training statistics

In Tables 5 and 6 we present the training times, number of training iterations and number of epochs that were required to train each system.

**Table 5:** Training statistics for the EN–DE NPE systems

System reference	Train time (minutes)	Iterations	# epochs
Vanilla 1	530	26,000	53
Vanilla 2	608	30,000	61
Vanilla 3	478	22,000	42
Vanilla 4	486	24,000	46
Vanilla 5	667	24,000	51
Vanilla 6	658	24,000	51
Vanilla 7	228	16,000	55
Vanilla 8	216	16,000	56
Augmented 1	690	24,000	50
Augmented 2	690	22,000	46
Augmented 3	699	26,000	54

The training of the EN–DE systems in the first round allowed us to conclude that the Transformer systems (Vanilla 7 and 8) were the fastest.

For the EN–ES round, we only trained and tested one of the Vanilla systems and the three augmented systems. The decision not to train all the same systems as for EN–DE was based on the first evaluations of the EN–DE systems. We present our results in Sect. 5.

**Table 6:** Training statistics for the EN–ES NPE systems

System reference	Train time (minutes)	Iterations	# epochs
Vanilla 1	549	28,000	40
Augmented 1	529	22,000	46
Augmented 2	528	22,000	46
Augmented 3	519	22,000	46

## Evaluation

Evaluation of the performance of the NPE systems, based on the translation of the test set, and its comparison to the PE it contained, was processed in several phases:

1. Collecting the standard metrics for APE evaluation: TER and BLEU, for all systems. TER was measured using the output of each NPE system as hypothesis, against the PE as reference. The presented BLEU scores are also computed on the premises that the NPE output is the hypothesis and the (human) PE is the reference. This data was first collected for EN–DE, and then for the EN–ES language pair only once the best EN–DE systems were evaluated (Sect. 5.1).
2. Then, we looked in detail at the results obtained by the different systems, focused on the precision of the systems, the number of edit operations, the results within the various partitions, and the minimum and maximum number of errors (Sect. 5.2).
3. Finally, an experimental evaluation of NPE applied to NMT output was conducted (Sect. 5.3).

### Standard evaluation scores

After training our systems, we evaluated them by computing BLEU and TER scores on the test sets. The results for EN–DE are presented in Table 7, and for EN–ES in Table 8.

**Table 7:** Evaluation scores for the EN–DE NPE systems

System reference	BLEU Moses		BLEU MultEval		TER MultEval	
	Dev	Test	Dev	Test	Dev	Test
Baseline	39.10	***41.07***	39.10	***40.80***	41.00	***39.20***
Vanilla 1	62.87	62.81	62.50	62.70	28.00	27.70
Vanilla 2	62.41	62.95	62.10	62.90	29.20	28.20
Vanilla 3	61.79	62.42	61.50	62.10	29.30	28.10
Vanilla 4	61.31	61.86	60.70	61.20	29.70	29.40
Vanilla 5	63.78	64.16	63.50	63.70	27.70	27.20
Vanilla 6	63.53	63.72	63.01	63.00	28.50	28.20
Vanilla 7	53.26	54.25	53.00	53.80	35.70	35.10
Vanilla 8	53.19	**53.91**	52.60	**53.20**	36.40	**35.50**
Augmented 1	64.38	64.87	64.10	64.50	27.60	26.70
Augmented 2	64.24	**65.01**	64.00	**64.60**	27.10	**26.20**
Augmented 3	64.47	64.99	64.20	64.60	27.10	26.30

For ease of readability baseline scores on the test set are highlighted in italic; among the NPE systems the worst scores (on the test set) are in bold font and underlined and the best scores are in bold

The first thing to observe from Tables 7 and 8 is that all tested NPE systems improve the quality of the translations of the baseline SMT output. These improvements are more visible in EN–DE, and the observations on the first round for this language pair helped us select a lesser number of systems for the second round for EN–ES. So, let us start by analysing the EN–DE scores.

For EN–DE, the minimum improvements were + 12.84, + 12.40 and − 3.70 in Moses BLEU, MultEval BLEU and TER, respectively, for system Vanilla 8. The maximum improvement is + 23.93, + 23.80 and − 12.90 in Moses BLEU, MultEval BLEU and TER, respectively, for the Augmented 2 system.

We also observe that multi-source systems outperform the concatenated ones (Vanilla 1 to 4). Two explanations for this are valid: (i) trimming of long sequences (longer than 300 tokens) removes tokens from the end of the concatenated input and as such the second part of the sequence may be significantly reduced; (ii) LSTMs are known to decay in performance the longer the input they are given, and even though Marian uses an attention mechanism that aims to tackle this problem, it has been shown that NMT decays in performance with the increase of the input length (Koehn and Knowles 2017). Concatenated approaches use the source as prefix and the SMT output as suffix; since these strings are sometimes trimmed, the second part of the sequence may be completely removed. We followed the approach of (Hokamp 2017) where SRC was the first string and the SMT the second. We ought to note that reversing the two, i.e. SMT first and SRC second, may impact the performance. However, in the two extreme cases where the second part is trimmed completely, the APE task is reduced to an MT task—either a monolingual (SMT to PE) or a bilingual (SRC to PE). Multi-source systems bypass the aforementioned problems and allow source and SMT to remain completely aligned.

One drawback of both NMT and NPE we picked in our experiments is that they deal with limited number of input tokens. When inputs to an NPE system are trimmed to fit such a limit it is unlikely that the output will be a correct post-edited version of the original MT. We acknowledge this limitation and will address it in our future work.

The results show that the best performing system for EN–DE is the one augmented with the TenantPartition token, i.e. Augmented 2. This system was selected as our best NPE system for EN–DE, and it was evaluated in-depth as reported in the following sections. This may mean that this token identifies the sets of strings that share more common features. This process permits a great deal of experimentation, by testing the addition of these tokens using different classes and methods.

Training speed is a particularly important feature in the evaluation of systems that are to be used in production settings. The Vanilla 7 and Vanilla 8 systems are the fastest during the training stages (see Table 5), because they use the ‘transformer’ approach. However, while these two transformer systems are approximately 3 times faster than the augmented ones (using a sequence-to-sequence method), the evaluation scores obtained by these systems in the same training circumstances are much lower. We admit the possibility that these systems might achieve a higher quality with a higher volume of training data, namely by using synthetic data (Junczys-Dowmunt and Grundkiewicz 2016; Chatterjee et al. 2018), but that was considered out of scope for this project.

**Table 8:** Evaluation scores for the EN–ES NPE systems

System reference	BLEU Moses		BLEU MultEval		TER MultEval	
	Dev	Test	Dev	Test	Dev	Test
Baseline	58.19	***60.09***	58.20	***60.10***	27.60	***25.60***
Vanilla 1	65.31	**66.06**	65.10	**65.80**	24.60	23.20
Augmented 1	66.07	**66.84**	65.90	**66.60**	23.70	23.20
Augmented 2	65.54	66.26	65.40	66.00	24.40	**23.50**
Augmented 3	66.19	66.78	66.10	66.50	23.30	**22.90**

For ease of readability baseline scores on the test set are highlighted in italic; among the NPE systems the worst scores (on the test set) are in bold font and underlined and the best scores are in bold

State-of-the-art APE systems can also deteriorate TER scores, especially because they over-correct the output of segments that require no editing (do Carmo et al. 2020). In our case, all NPE systems yielded improvements in the global scores, which shows the potential of the NPE approach. It is important to stress this, since, for EN–ES, this was achieved with very high BLEU and very low TER scores in the initial SMT output, a circumstance in which it would be very difficult to get any improvements.

As might be predicted, due to the already high initial values of BLEU and TER in the training data, the improvements in the scores were not so visible for EN–ES, and the intervals between systems were much narrower. In this language pair, there was no clear system with the best scores for both metrics: Augmented 1 (trained with Length as the added token) obtained the best scores in BLEU (66.84 as measured by Moses, and 66.60, as measured with MultEval), but Augmented 3 (trained with Tenant as the added token) achieved the best TER score (22.90). This is a very different outcome from EN–DE, in which it had been one system trained with the TenantPartition token to outperform all others, according to the three metrics. Since we are using TER as the main metric, we chose Augmented 3 as our best system for EN–ES. The improvements from the SMT output obtained by this system were of a mere 2.7 TER points and less than 7 BLEU points, much lower values than those obtained in EN–DE.

### Detailed analysis of editing results

This detailed analysis tried to answer two questions: (i) how good are the best NPE systems and (ii) for which input is NPE better. To answer (i) we measured the precision of the NPE systems, and we analysed the balance in the distribution of edit operations. To know on which type of input NPE achieves the best results, we looked into the distribution of edit operations, per each major data partition, and we analysed the behaviour of very long and very short segments, besides those that required the least number of edits and those that required the maximum number of edits.

*Precision* This is measured as a ratio of the segments in which TER was improved over the total number of segments that were modified. To measure this precision, we identified the segments in which there were content differences between SMT and PE, since these were the segments modified by the NPE system. Then, we compared the TER(smt,pe) and TER(npe,pe) in those segments, and we considered as improved those segments in which the TER of the NPE was lower than the one for SMT. We report the number of improved and deteriorated segments, and the number of those that were edited by the NPE system, but which resulted in the same TER.

As we can see in Tables 9 and 10, the precision metric confirms the capacity of the NPE system to improve the global TER of the test sets. In EN–DE, 76% of the sentences in the test set were modified, and a number that corresponds to 62% of these was improved. The EN–ES NPE seems to be more conservative, only modifying 67% of the segments, and less precise, since only 51% of the modified segments were improved. The best system in EN–ES also deteriorated a higher number of sentences (35%, against 25% in EN–DE). The percentage of segments which were modified with no effect on TER was fairly similar in both language pairs (12% in EN–DE and 14% in EN–ES).

**Table 9:** Precision of best NPE system for EN–DE

	Description	No.	%
Total segments in test set		10000	
Non-modified segments	SMT=NPE (content)	2396	24
Modified segments	SMT$\ne$NPE (content)	7604	76
Improved segments	TER(npe, pe)<TER(smt, pe)	4734	62
Deteriorated segments	TER(npe, pe)>TER(smt, pe)	1923	25
Segments with same TER	TER(npe, pe)=TER(smt, pe)	947	12

**Table 10:** Precision of best NPE system for EN–ES

	Description	No.	%
Total segments in test set		10000	
Non-modified segments	SMT=NPE (content)	3350	34
Modified segments	SMT$\ne$NPE (content)	6650	67
Improved segments	TER(npe, pe)<TER(smt, pe)	3415	51
Deteriorated segments	TER(npe, pe)>TER(smt, pe)	2323	35
Segments with same TER	TER(npe, pe)=TER(smt, pe)	912	14

*Distribution of edit operations.* In Tables 11 and 12 we compare the best NPE systems to the baseline SMT output, in terms of the edit operations that result in the distances between these outputs and the original PE versions.

**Table 11:** Number of edit operations in SMT and NPE for EN–DE

	Insertions	Deletions	Substitutions	Shifts	Total
SMT: TER(smt, pe)	3526	14,795	17,427	4587	40,335
NPE: TER(npe, pe)	4136	7717	12,860	2404	27,117
(NPE-SMT)	610	− 7078	− 4567	− 2183	− 13,218

**Table 12:** Number of edit operations in SMT and NPE for EN–ES

	Insertions	Deletions	Substitutions	Shifts	Total
SMT: TER(smt, pe)	4745	6213	14,308	3057	28,323
NPE: TER(npe, pe)	3543	7974	11,947	1888	25,352
(NPE-SMT)	− 1202	1761	− 2361	− 1169	− 2971

The NPE system for EN–DE would require far fewer edit operations to be transformed into the PE than the SMT system. This reduction in the number of edit operations affected all types of edits except insertions, which the NPE system would require more than the SMT system. This result is very different for the EN–ES systems. In this case, the number of edits is also lower for the NPE system than for the SMT system, but the reduction is much smaller. Besides, instead of more insertions, the NPE makes more deletions than the SMT system, but again the difference is not very relevant.

**Fig. 2:**
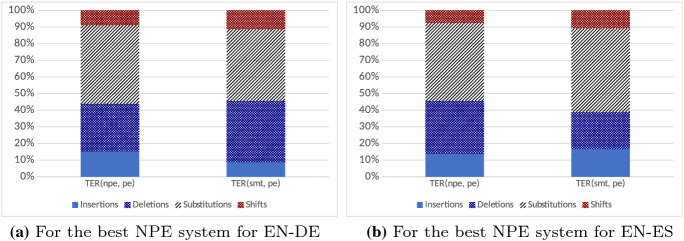
Distribution of edit operations in both language pairs

Figure 2 shows that the distribution of edit operations is well-balanced, in both language pairs. Substitutions are the most frequent edit, representing almost 47% of the total, followed by deletions (28% and 31% for EN–DE and EN–ES, respectively), with insertions at around 15%, and shifts below 10%.

We also assessed the most frequently edited tokens by the EN–DE best system. The top 5 most frequently edited tokens per editing operation are:

- *Insertions* The five most frequently inserted tokens account for 14% of all insertions, two of them being the words “Sie” (5.8% of all insertions) and “Bitte” (1.8%). The other three more frequently inserted tokens are punctuation and placeholders.
- *Deletions* The five most frequently deleted tokens account for almost 20% of all deletions. The most frequently deleted tokens are punctuation (comma 6.4%, neutral double quote 5.6% and full stop with 2.4%), the pronoun “Sie” (3.0%) and the article “die” (2.3%).
- *Substitutions* Substitutions are much more sparse; the five most frequent substituted tokens only account for 2.3% of all substitutions. Punctuation tokens constitute a major part of the substitutions, but the most frequent one is capitalisation—8.2% of all substitutions.

The next question we tried to answer was: in which type of segments is NPE most effective? To help answer this, we analysed our data partitions, besides the original TER partitions, and the maximum and minimum types of segments.

*Length partitions* In Table 13 we show the average TER scores in each length partition for both language pairs, obtained by the best NPE systems. We can see that the common result is that the lowest average TER scores (i.e. the best) are obtained in the mid-length segments (between 5 and 30 words). The shortest segments (below 5 words) seem to be the ones in which the NPE systems have more difficulties in achieving good TER scores, in both language pairs.

**Table 13:** Average TER scores per length partition, for both language pairs

Length partitions	EN–DE	EN–ES
0-4	35.50	29.55
5-9	23.69	19.92
10-30	23.82	21.90
31-300	29.73	24.78

*TenantPartition* The TenantPartition groups tenants in six classes. We investigated whether there were differences in the TER scores obtained after NPE among these classes. In this case, the biggest differences came from the comparison between the TER scores of the SMT and the NPE systems.

Tables 14 and 15 show how *Chicago* and *Exchange15* are the two tenants that achieve the highest reduction in TER scores, from SMT to NPE output, for both language pairs. In contrast, *OMain2* was, for both language pairs, the tenant in which the TER scores, on average, increased the most.

We can also see that, for both SMT and NPE output, and in both language pairs, *OMain2* is always the tenant in which we get the lowest TER scores. However, we did not find a correlation between the highest/lowest TER scores and the reduction/deterioration in the TER scores between the baseline SMT and the NPE systems, in this analysis per TenantPartition.

These results show the relevance of using the tenants as informative tokens for NPE, as we had seen for length. Since these tenants probably group different types of content, this analysis could be further explored in future studies, by looking at the specific linguistic features that create this difference in results.

**Table 14:** Average TER scores per TenantPartition of SMT and NPE for EN–DE

TenantPartition	Avg TER(smt, pe)	Avg TER(npe, pe)	Reduction
Chicago	44.64	29.94	− 14.70
OMainCCRel	45.80	37.39	− 8.40
Exchange15	41.65	25.12	− 16.53
OMain2	19.10	22.15	3.06
BitHum	39.31	41.53	2.22
Other	37.28	30.79	− 6.49

**Table 15:** Average TER scores per TenantPartition of SMT and NPE for EN–ES

TenantPartition	Avg TER(smt, pe)	Avg TER(npe, pe)	Reduction
Chicago	29.66	25.22	− 4.44
OMainCCRel	29.49	26.70	− 2.79
Exchange15	28.50	22.95	− 5.55
OMain2	13.17	19.84	6.67
BitHum	29.23	33.55	4.32
Other	23.81	26.36	2.55

*TER ranges* We then investigated how the best NPE systems performed according to the initial TER scores of the SMT output. We grouped the segments in the test set according to ranges of 20 TER points, we counted the number of segments in those ranges in SMT and in NPE output, and then we estimated the percentage of those counts over the whole test set. The observations are illustrated in Tables 16 and 17.

**Table 16:** Number and percentage of segments per TER reange in SMT and NPE output for EN–DE

TER ranges	SMT	% total	NPE	% total	Diff.
0	2947	29	4076	41	1129
1–19	659	7	1214	12	555
20–39	1858	19	1602	16	− 256
40–59	2013	20	1170	12	− 843
60–79	1214	12	604	6	− 610
80–99	298	3	162	2	− 136
100–1140	1011	10	1172	12	161

**Table 17:** Number and percentage of segments per TER range in SMT and NPE output for EN–ES

TER ranges	SMT	% total	NPE	% total	Diff.
0	3782	38	3910	39	128
1–19	1442	14	1724	17	282
20–39	2219	22	2015	20	− 204
40–59	1273	13	982	10	− 291
60–79	613	6	480	5	− 133
80–99	164	2	122	1	− 42
100–1140	507	5	767	8	260

Tables 16 and 17 show an increase in the number of segments with TER scores in the first two ranges, of zero TER and of scores up to 20 points, for both language pairs, after NPE was applied. In EN–DE, the percentage of segments with zero TER increased from 29% in the SMT output to 41% in NPE output. In EN–ES, the initial number of segments in this TER range in SMT was already high, 38%, but this increased one point, to 39% in NPE. The two first ranges gained in total 1684 segments in EN–DE, and only 410 segments in EN–ES.

The only range in which this percentage increased again was in the number of segments with TER scores above 100, an increase that reflects a deterioration in TER scores caused by NPE. It is important to note that these are segments in which all words are edited or there is an addition of words in the edited version. (Since TER is not capped, if an edited sentence has more words than the reference, then the score will be higher than 100%.) There is a long tail of segments in this range, in both outputs and in both language pairs. In EN–DE, the number of segments in this highest TER range (of 100–1140 TER points) increased from 10 to 12%, and in EN–ES from 5 to 8%.

The number of segments in the four TER ranges between these two extremes (segments with TER between 20 an 99%) always decreased from SMT to NPE. The TER scores of the sentences in these ranges could have improved or deteriorated, but we can see that the vast majority of these sentences saw their TER scores reduced to a value between 0 and 19. Let us look in detail at the table for EN–DE, for example. There are 161 segments that had a TER below 100 in SMT and whose score was deteriorated to values between 100-1140% by NPE. We can assume that most of these segments belonged to the 80-99 range: this range lost 136 segments, and we only need another 25 segments from the 60 to 79 range to have all segments in which the TER scores deteriorated. This means that NPE was able to reduce the TER to a value between 0 and 19% editing in a number of sentences that is equivalent to those that had editing scores from 20 to 79% but which moved to a different 20-point range of editing scores.

Our observations thus suggest that NPE systems are capable of reducing the TER score obtained by SMT systems, measured against the human PE, in segments that show a wide variation of initial TER scores. However, these systems show bigger difficulties at higher levels of editing. Of course, we are only looking at gross numbers of segments between 20-point ranges; many changes in the TER scores happened within the ranges that we use in this analysis. We look next at some of the extreme TER scores in the dataset.

*Segments with extreme editing* The final task in this detailed evaluation looked at segments with very low or very high numbers of errors, as identified by TER, and for very long and very short segments.

TER(npe, pe) counts as an error, or edit, every word that has been deleted, inserted, substituted or shifted in the NPE translation, when compared to the PE. Table 18 shows the number of segments with zero edits in the SMT output, the number of segments with zero edits in the NPE output, and the intersection between the two columns, i.e. the number of segments which have zero edits in both outputs. In EN–DE, of the ca. 3000 segments that had zero errors (no edits when compared to PE) in the SMT output, only ca. 2000 (i.e. 67%) were maintained in that condition. The rest will have been edited somehow by the NPE and their TER was deteriorated. However, the number of segments with zero edits in NPE was increased by an extra 1000. This same tendency is seen in segments with one error, in this case with different percentages. This means that, although NPE does deteriorate the TER in a portion of the best scores from SMT, it is capable of improving the global TER scores by reducing the TER in a higher number of segments.

In the other extreme, for segments with a high number of errors, above 100 (and this number reaches 382 in one single segment), there is a higher number of segments in NPE than in SMT. The seven segments in this group have all more than 100 words, they include several sentences, and some are composed of long lists of non-translatable code, placeholders, and similar UI content. NPE truncated some of these segments, it translated non-translatable elements, and it has missed the detailed translation required within these segments. NPE edited almost all of the words in these segments, which justifies that there are 1700 errors in 2100 words in these segments alone. We may add that in all 290 segments with more than 100 words, only five have less than 100 errors, but that all segments with more than 200 words are in this list of segments with more than 100 errors. Considering the magnitude of errors added to the TER scores in these sentences alone, a process that tackled this problem alone could have a big effect in the precision of the system. As alluded for in the data analysis (Sect. 3.1), this type of issue could be solved with a pre-processing stage to split long segments that include several sentences into single-sentence segments.

**Table 18:** Number of segments with minimum and maximum editing in SMT and NPE output for EN–DE

Errors per segment	SMT	NPE	Both	% SMT
NumErr = 0	2947	4076	1983	67
NumErr = 1	1311	1787	504	38
NumErr > 100	2	7	0	
Total errors in seg NumErr>100	290	1700	0	

Table 19 reports the equivalent data for the EN–ES NPE systems, and the behaviour is similar.

**Table 19:** Number of segments with minimum and maximum editing in SMT and NPE output for EN–ES

Errors per segment	SMT	NPE	Both	% SMT
NumErr = 0	3782	3910	2566	68
NumErr = 1	1400	1887	620	44
NumErr > 100	0	7	0	
Total Errors in seg NumErr>100	0	1154	0	

*Segments with extreme lengths* The analysis of the shortest and longest segments, summarised in Tables 20 and 21, shows another dimension of the need for some form of optimisation for specific segments.

**Table 20:** Number of segments with minimum and maximum numbers of words in SMT and NPE output for EN–DE

Short and long segments	No.	SMT_TER	NPE_TER	Reduction
One-word segments	1351	27.4	56.07	28.67
Two-word segments	1794	38.52	32.94	− 5.58
Segments with +100wds	12	28.07	44.1	16.03

**Table 21:** Number of segments with minimum and maximum numbers of words in SMT and NPE output for EN–ES

Short and long segments	No.	SMT_TER	NPE_TER	Reduction
One-word segments	1130	22.02	50.42	28.4
Two-word segments	1598	28.52	29.25	0.73
Segments with + 100 wds	15	20.2	51.96	31.76

The edit scores in one-word segments are heavily deteriorated by NPE systems, for both languages. If we look in detail to these segments, we will see some of the effects of “hallucinated” BPE words. In two-word segments, the scores improve, and in EN–DE NPE already achieves a better editing score than SMT. In EN–ES, the improvement is not so high to make NPE improve on the SMT. In the long segments, as we have seen above, the deterioration of TER scores by NPE is much higher. As we mentioned before, the longest segments include several strings of localisation code and placeholders, elements which make it very hard for any MT systems to identify the language content and translate it adequately. We can see from this brief analysis that NPE is well tuned to mid-length segments in such a way that it compensates for this lack of capacity in segments at the length extremes.

*Summary of detailed evaluation* This detailed evaluation has answered the two questions we initially asked. First, it has shown that our best NPE systems in each language pairs were very precise and also balanced, in terms of the distribution of edit operations. Besides, it helped us see that the added tokens of length and tenant were in fact very informative, since they pointed to partitions of data in which, although the distribution of TER improvements was not equal, the gains in some of the partitions compensated for the losses in others. Finally, this evaluation allowed us to identify types of segments in which TER deterioration occurred most frequently, namely segments which show more than 100 errors in SMT output and those that have one, two or more than 100 words, information which can help devise methods to tackle this specific deterioration and thus improve the precision of an NPE system to be used with this type of data.

### Evaluation of NPE on NMT output

After the global evaluation of the performance of all NPE systems, the best performing systems were applied to NMT output. For EN–DE, we used the Augmented 2 system, trained with the TenantPartition token. For the EN–ES language pair, we use Augmented 1, trained with the Length token, as explained before.

We applied our NPE on custom NMT systems (sequence-to-sequence with LSTM units), trained with Marian-NMT, with default parameters for EN–DE and EN–ES. For training we used in-domain data after we ensured that there were no overlaps with the APE training data. We then translated the original test set source strings and next we applied our NPE systems. The output of these systems is referred to as “NMTcustom with NPE” in Tables 22 and 23. The performance of this system was measured against the original PE reference.

In industry it is still the case that SMT rather than NMT systems proliferate in real production scenarios, as these SMT systems have continuously been updated and tuned to achieve high performance in the actual translation pipelines deployed. While NMT systems continue to improve, and despite the fact that those which are used in production have surpassed the quality obtainable via SMT, our main objective in these experiments was to show that while NPE can improve NMT output, the gain is lower than when post-editing SMT output. Besides, at the time of this project (2018), the amount of post-edited NMT data was not adequate to fully train and test the learning capacity of an NPE system applied to NMT output.

We can see from Tables 7 and 8 that these are under-performing NMT systems, with TER and BLEU scores that do not represent major improvements from the baseline SMT systems. This may be due to big differences between the training data used for the NMT and the actual data in the test set. Still, this created the conditions to test the application of the lessons learnt by the NPE system on SMT output to new NMT output.

The NPE systems (having been trained on SMT output), receive the SRC+NMT output of the test set as multi-inputs, and translate this input into an improved version in the target language.

It is important to note that this approach is against the assumptions of an APE process: the editing patterns that have been learnt during training are closely dependent on the output of the MT system used originally, in this case, SMT systems, so the use of input from a different MT system may present unpredictable results. Still, these results contribute to answering a different question: can an APE system trained on SMT output be used to improve NMT output?

**Table 22:** Comparison of custom NMT system before and after NPE for EN–DE

Sys. ref	BLEU (Moses)	BLEU (MultEval)	TER (MultEval)
NMTcustom	41.51	41.20	39.10
NMTcustom with NPE	59.11	58.80	31.90
(Auggmented 2)

**Table 23:** Comparison of custom NMT system before and after NPE for EN–ES

Sys. ref	BLEU (Moses)	BLEU (MultEval)	TER (MultEval)
NMTcustom	59.71	59.40	24.40
NMTcustom with NPE	62.07	61.90	26.70
(Auggmented 1)			

We can see that, for EN–DE, TER has been reduced in − 7.20, and BLEU increased 17.60, when we applied NPE to an underperforming NMT system output. For EN–ES, there were visible improvements in BLEU (2.50), but TER deteriorated (+ 2.30).

A conclusion we can take from these results is that NPE systems are not able to achieve as high improvements in NMT output, as they can when working with outputs from different MT technologies. Besides, although the EN–DE results seem to be promising, the results in the other language pair seem to fail, in view of a thinner learning margin. Further experiments, and a detailed analysis of the results, would be required to give a more accurate answer to the question of the applicability of an NPE system trained on SMT output to improve NMT output.

## Conclusions

This article presents the outcome of a joint project between ADAPT and Microsoft on the topic of Neural Post-Editing (NPE). We investigated different options for building an NPE system, drawing a roadmap to aid with the major decision points. This map covers decisions about preprocessing (segmentation and tokenisation) of the data, the NPE architecture, the approach chosen to present the input/output to the system, as well as how to introduce extra information, targeting optimal performance. Following this roadmap, we identified the optimal setup—multi-source LSTM encoder/decoder networks with data augmented with extra information (presented as a prefix token, attached to each triplet) for both EN–DE and EN–ES. The use of prefix tokens has proven beneficial in MT for domain adaptation, for correcting politeness, and for improving gender issues. To our knowledge, this work was the first to use this approach for automatic post-editing, and has been shown to lead to the best performing systems.

In addition, we conducted an in-depth comparative analysis on the trained systems with respect to TER. The effects of our systems have been positive under all investigated criteria. Furthermore, the detailed evaluation of the results showed areas in which research can invest to improve or fine-tune NPE application to specific use cases.

This project has demonstrated a positive contribution from APE in improving the output of MT content production workflows in commercial environments. Companies dealing with translation data over the years have accumulated post-edited data which may be tapped into and used to learn editing patterns that may help improve the outputs of MT systems. This contribution is not so clear when content production systems are based on NMT, so more research is needed to clarify how APE can improve on this output.

One issue that deserves further attention is related to how sequence-to-sequence systems, and in our case APE systems, handle long inputs, e.g., beyond our 300-token limit. In an industry setting, this can be common for many texts. In the future, we would like to investigate this issue and we consider paragraph or document level APE with smart ways to segment longer input sequences.

## Footnotes

1 We refer to these scores as TER instead of HTER since we use the PEs as references with no further human post-editing. That is, it is simply an automatic scoring of TER between a hypothesis (output by an MT or an APE system) and a reference (produced by a human translator).
